# Transbronchial lung cryobiopsy for the diagnosis of diffuse parenchymal lung disease: Pitfalls and challenges, a single center experience

**DOI:** 10.55730/1300-0144.5563

**Published:** 2022-08-04

**Authors:** Kemal Can TERTEMİZ, Aylin Özgen ALPAYDIN, Nurcan GÜLER, Volkan KARAÇAM, Duygu GÜREL, Naciye Sinem GEZER

**Affiliations:** 1 Department of Respiratory Diseases, Dokuz Eylül University Faculty of Medicine, İzmir, Turkey; 2 Department of Thoracic Surgery, Dokuz Eylül University Faculty of Medicine, İzmir, Turkey; 3 Department of Pathology, Dokuz Eylül University Faculty of Medicine, İzmir, Turkey; 4 Department of Radiology, Dokuz Eylül University Hospital, İzmir, Turkey

**Keywords:** Transbronchial lung cryobiopsy, interstitial lung disease, diagnosing

## Abstract

**Background/aim:**

Transbronchial lung cryobiopsy (TBLC) is a minimally invasive technique of the diagnosis of diffuse parenchymal lung diseases (DPLD). The aim of this study is to determine the clinical-radiological and histopathological characteristics of patients in whom cryobiopsy contributes to the diagnosis.

**Materials and methods:**

In this retrospective study, we searched for the medical records of patients who underwent TBLC from July 2015 to March 2020 at the pulmonology department of our university hospital clinic. Radiological images were evaluated by a chest radiologist experienced in DPLD. Prediagnosis was indicated by clinical-radiological findings. The final diagnosis was determined by the contribution of histopathological diagnosis. The agreement of pretest/posttest diagnosis and the diagnostic yield of TBLC were calculated.

**Results:**

Sixty-one patients with female predominance (59.0%) and current or ex-smoker (49.2%) made up the study population. We found the diagnostic yield of TBLC 88.5%. The most common radiological and clinical-radiological diagnosis was idiopathic pulmonary fibrosis (IPF) (n = 12, 19.6%) while the most common multidisciplinary final diagnosis was cryptogenic organizing pneumonia (COP) (n = 14, %22.9). The concordance of pre/posttests was significant (p < 0.001) with a kappa agreement = 0.485. The usual interstitial pneumonia (UIP) diagnosis was detected in six patients among 12 who were prediagnosed as IPF having also a suspicion of other DPLD by clinical-radiological evaluation (p < 0.001). After the contribution of TBLC, the multidisciplinary final diagnosis of 22(36.1) patients changed. The histopathological diagnosis in which the clinical-radiological diagnosis changed the most was nonspecific interstitial pneumonia (NSIP).

**Conclusion:**

We found the overall diagnostic yield of TBLC high. The pretest clinical-radiological diagnosis was often compatible with the multidisciplinary final diagnosis. However, TBLC is useful for the confirmation of clinical radiological diagnosis as well as clinical entities such as NSIP which is difficult to diagnose clinical-radiological. We also suggest that TBLC should be considered in patients whose clinicopathological IPF diagnosis is not precise.

## 1. Introduction

Diffuse parenchymal lung diseases (DPLD) are a heterogeneous group of lung disorders, comprising more than two hundred diseases [1]. DPLD pathogenesis is characterized by alveolar epithelial cell injury, inflammation, and parenchymal fibrosis [2]. The recommended approach for the diagnosis of DPLD is the multidisciplinary evaluation of the clinician, radiologist, and pathologist [3].

The widespread and effective use of antifibrotic treatments in idiopathic pulmonary fibrosis (IPF), an important and relatively common entity among DPLD, has increased the importance of definitive diagnosis. Histopathological diagnosis is recommended in DPLD patients who cannot be diagnosed with clinical and radiological findings [4].

Transbronchial lung cryobiopsy (TBLC) is a minimally invasive technique alternative to surgical lung biopsy (SLB) in the diagnosis of DPLD [5,6]. The diagnostic yield of TBLC has been found to be high (pooled estimate of 83% (95% confidence interval [CI], 73–94), however, complication rates vary in a wide range (pooled estimates for pneumothorax and moderate/severe bleeding were 12% (95% CI, 3–21) and 39% (95% CI, 3–76), respectively) [7]. TBLC is recommended in a specific group of DPLD patients where the integration of clinical and high-resolution computed tomography (HRCT) features is not sufficient to make a definitive diagnosis. In addition, TBLC may be performed if a clinical diagnosis other than IPF is suspected, even if HRCT findings are consistent with typical usual interstitial pneumonia (UIP) [8]. Although there are many studies covering this issue, the reliability of TBLC in the DPLD diagnostic algorithm has not been determined definitively [9–11].

Recent studies and guidelines have clearly revealed the indications for TBLC [4–7]. In our clinical experience, TBLC has provided useful information to well selected patients with DPLD. The aim of this study is to determine the characteristics of patients to whom TBLC contributed to the differential diagnosis of DPLD.

## 2. Materials and methods

### 2.1. Study design

In this retrospective study, the medical records of patients who underwent TBLC in the pulmonary diseases department of our university hospital between July 2015 and March 2020 were scanned from the electronic hospital database. The local ethics committee approved this study with protocol number 2021/05-26.

### 2.2. Patients

**Figure 1:**
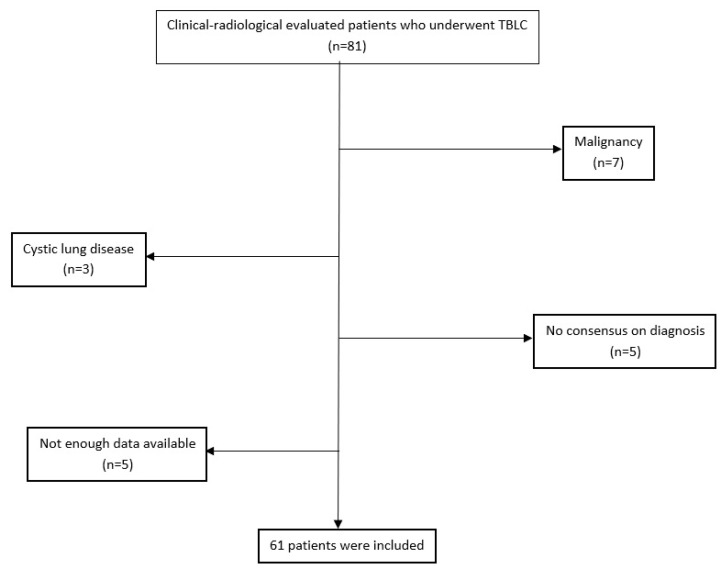
Flow chart of the study population inclusion. TBLC: Transbronchial lung cryobiopsy

Eighty-one patients were screened. The indications for TBLC were the presence of clinical suspicion of other diagnoses despite a typical UIP pattern in HRCT and where differential diagnosis could not be made by clinical and radiological multidisciplinary evaluation. Exclusion criteria were patients under 18 years of age and TBLC for indications other than DPLD. Finally, we identified 61 patients to be included in the analysis (Figure 1).

The demographic characteristics of the study population such as age, sex, smoking status, medication, occupational history, lung functions, TBLC procedure details, complications, and histopathological evaluation were recorded from the electronic database of the hospital.

### 2.3. CT scanning protocol and interpretation

HRCT scans were obtained with one of two different multidetector CT scanners (Brilliance 64 Philips, Brilliance 16 Philips, and MX 8000 Philips; Philips Medical Systems ©, Eindhoven, The Netherlands) using a standardized protocol. Images were acquired using thin-section (1.25 mm) volumetric HRCT with 120 kV, 285 mA, and 1 to 2 s scanning time during breath holding at end inspiration. A high spatial frequency algorithm was used, and images were read at window levels appropriate for pulmonary parenchyma evaluation (level 600 to 700 Hounsfield units; window 1500–1600).

A thoracic radiologist with more than 10 years of experience (N.S.G.) who was blinded to all patient information evaluated the HRCT scans for the presence of the following elements: reticulations, interface sign, honeycombing, cysts, emphysema, traction bronchiectasis or bronchiectasis, peribronchial thickening, ground-glass opacification, consolidation, mosaicism, and nodules (centrilobular, perilymphatic or random patterns). Information pertaining to the distribution of the abnormalities was also evaluated. The presence of heterogeneity was evaluated in the axial plane while the presence of apicobasal gradient or upper zone predominance was evaluated in the coronal plane.

Interstitial fibrosis in HRCT was classified according to the current diagnostic criteria of the Fleischner Society for IPF [12]. The radiologist was asked to make a final diagnosis based on imaging findings only. The diagnosis of radiologists included idiopathic pulmonary fibrosis (IPF), nonspecific interstitial pneumonia (NSIP), hypersensitivity pneumonitis (HSP), respiratory bronchiolitis (RB), lymphocytic interstitial pneumonia (LIP), desquamative interstitial pneumonia (DIP), organizing pneumonia (OP), pleuroparenchymal fibroelastosis (PPF), lymphangioleiomyomatosis (LLM), langerhans cell histiocytosis (LHH), vasculitis, sarcoidosis, malignancy, and diffuse alveolar hemorrhage (DAH). Subsequently, assessments were performed by a multidisciplinary team of a radiologist and two independent blinded pulmonologists, and a clinical-radiological diagnosis was made for each patient based on both imaging findings and clinical information.

### 2.4. TBLC procedure

The TBLC procedure was performed as described previously [10]. Following deep sedation with intravenous propofol and remifentanil patients were intubated with a rigid video bronchoscope (TEXAS Optical Fully Integrated Rigid Bronchoscope, Houston, TX, USA). Fluoroscopic guidance was used for the procedure and the biopsies were obtained using a flexible cryoprobe (2.4 mm; ERBE, Tübingen, Germany). Before the procedure, the bronchoscopist planned where to take TBLC according to the HRCT image of each case. During the procedure, the Fogarty balloon occlusion catheter was positioned at the targeted segmental bronchial inlet in all cases. The mean freezing time for tissues taken by TBLC was 6–8 s.

Contraindications for TBLC were pulmonary systolic arterial pressure >40 mmHg on echocardiography, coagulopathy (platelet count <70.000/μL and/or prothrombin time international normalized ratio >1.5), hemodynamic instability, and severe hypoxemia (partial arterial oxygen pressure ≤55 mmHg on room air).

### 2.5. Histopathology

The final diagnosis was made with a multidisciplinary evaluation by evaluating the clinical and radiological findings together with the histopathology results obtained by TBLC.

## 3. Statistical analysis

Statistical analyses were performed using the SPSS (Statistical Package for Social Sciences) 24.0 software package. Descriptive data were given as mean and standard deviation (SD) or median and interquartile range (IQR). Categorical variables were expressed as the number of cases and the percentage value. The comparison of categorical variables was performed using chi-square and Fisher’s exact tests. Pre and posttest agreements were analyzed by Cohen’s kappa analysis. Statistical significance was set as p < 0.05.

## 4. Results

In this study, 61 patients with a prediagnosis of DPLD were analyzed. The flow chart of the inclusion procedure is shown in Figure 1. The baseline characteristics of the study population were as follows: relatively young patients (53.3 ± 11.5 years) with female predominance (59.0%) and a history of current or ex-smoking (49.2%) in almost half. The mean forced expiratory volume in 1s (FEV1) and forced vital capacity (FVC) values were relatively preserved, however, there was a decrease in the diffusing capacity for carbon monoxide (DLCO). The mean body mass index of the patients was 27.2 (18.9–42.0) kg/m^2^. There were three patients with a body mass index greater than 35 kg/m^2^. TBLC complications occurred in 22.9% of the patients (n = 14); hemorrhage was the most common that was massive in four (6.5%). During the 30-day postprocedural period there were no deaths and no disease exacerbations. Only one patient was hospitalized for subcutaneous emphysema on the fourth day after the biopsy and treated with nasal oxygen. Table 1 shows the baseline characteristics of all the study population.

**Figure 2:**
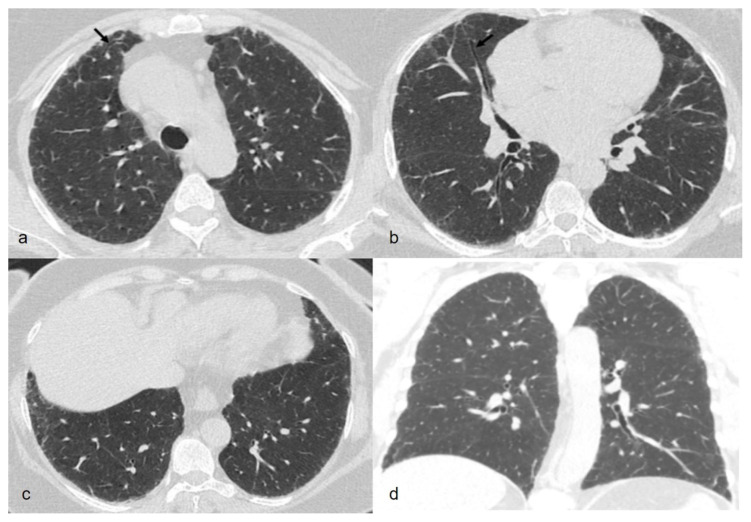
Axial slices of high-resolution computed tomography (HRCT) images in a 63-year-old patient demonstrate bilateral and peripheral fine reticulations, mild ground glass opacities and traction bronchiectasis (arrows) without any lober predominance (Figures 2a–2c). Middle and lower lobe predominancy or peribronchovascular distribution associated with organizing pneumonia are not detected in coranal slice of HRCT (Figure d). The patient was classified as nonspecific interstitial pneumonia, however the histopathologic diagnosis was organizing pneumonia.

The most common HRCT findings were ground glass opacification (n = 49, %80.3), reticulations (n = 41, 67.2%), traction bronchiectasis or bronchiolectasis (n = 35, 57.3%) and interface sign (n = 26, 42.6%). Other radiological findings demonstrated were nodules (n = 24, 39.4%), peribronchial thickening (n = 18, 29.5%), consolidation (n = 17, 27.9%), emphysema (n = 12, 19.7%), honeycombing (n = 11, 18.0), mosaicism (n = 10, 16.4%), cysts (n = 6, 9.8%). The nodules were in the form of the centrilobular pattern (n = 15, 62.5%), perilymphatic pattern (n = 4, 16.6%), random distribution pattern (n = 4, 16.6%), centrilobular and distribution pattern together (n = 1, 4.2%). According to these HRCT findings dominant radiological patterns were UIP (n = 10, 16.3%), OP (n = 9, 10.7%), RB (n = 7, 11.4%), NSIP (n = 4, 6.5%), cystic lung (n = 2, 3.2%) and tree-in-bud sign (n = 2, 3.2%). HRCT scans of a patient radiologically classified as NSIP but histopathologically diagnosed as OP is shown in Figures 2a, 2b, 2c, and 2d.

According to Fleishner’s radiological IPF classification, most of the patterns were alternative diagnostic HRCT patterns (n = 36, 59.0%). Usual interstitial pneumonia (UIP), probable UIP and indeterminate patterns were observed in 9 (14.7%), 9 (14.7%) and 7 (11.4%) of the patients, respectively. The presence of alternative diagnosis patterns such as heterogeneity, apicobasal gradient, and upper zone predominance were detected in 26 (42.6%), 32 (52.4%), and 11(18.0%), respectively.

The number of biopsies taken during the procedure was three (n = 40, 65.5%) in the majority of patients, with a minimum of one and a maximum of five. The most common site of TBLC was the lower lobe (right side n = 53, 86.8%, left side n = 6, 9.8%).

TBLC was performed from three segments in 65.5% of patients (n = 40), two segments in 18.0% (n = 11), four segments in 14.7% (n = 9) and one segment in 2.0% (n = 3). All biopsies were taken from a single lobe. The mean size of the biopsies was 5.7 (3–18) mm.

TBLC was nondiagnostic in seven patients, and the diagnostic yield of TBLC was 88.5% (n = 54). Among the patients with histopathology of normal parenchyma, one patient underwent SLB and was diagnosed with hypersensitivity pneumonia. SLB was planned for another patient to distinguish vasculitis and miliary tuberculosis and the results were similar to TBLC.

Among the patients whose tissue histopathology obtained by TBLC was interpreted as normal parenchyma, one was diagnosed with hypersensitivity pneumonia after a surgical lung biopsy (SLB). SLB was planned for another patient to distinguish vasculitis and miliary tuberculosis and the histopathological diagnosis was like that obtained by TBLC.

According to clinical presentation, HRCT, and histopathologic findings, clinical-radiological, radiological, and multidisciplinary final diagnoses including histopathology were assessed (Table 2). The most common radiological and clinical-radiological diagnosis was IPF (n = 12, %19.6), followed by cryptogenic organized pneumonia (COP) (n = 10, 16.3%), while the most common multidisciplinary final diagnosis was COP (n = 14, 22.9%). Among the patients who were not considered as IPF (n = 52) by multidisciplinary final diagnosis were OP, NSIP, respiratory bronchiolitis-interstitial lung disease (RBILD), granulomatous inflammation, pneumoconiosis, eosinophilic pneumonia, vasculitis, LHH, connective tissue disease-interstitial lung disease (CTD-ILD), hemosiderosis, and alveolar hemorrhage.

When clinical-radiological diagnosis (pretest) and histopathological diagnosis (posttest) were compared, the histopathological UIP diagnosis was detected in six patients among 12 who were clinical-radiological prediagnosed as IPF. In patients prediagnosed as non-IPF only three patients (6.2%) had histopathological UIP patterns. The concordance of pre and posttests were significant (p < 0.001), and kappa agreement = 0.485 (p < 0.001) (Table 3).

After the contribution of TBLC, the multidisciplinary final diagnosis of 22 (36.1%) patients had changed. For the remaining 32 (52.5%) (excluding seven nondiagnostic histopathologies) the clinical-radiological and histopathological assessments were compatible. IPF (n = 6 %50) and HSP (n = 4, 100%) were the most common diagnosis that had changed. The histopathological diagnosis in which the clinical-radiological diagnosis changed the most was NSIP. The details of the patients whose final diagnosis changed with TBLC are listed in Table 4.

## 5. Discussion

TBLC is a trending procedure that has been widely used for lung tissue sampling in the diagnosis of DPLD in recent years. In this study, we aimed to determine the contribution of TBLC to the diagnosis of DPLD. We found that the diagnostic yield of TBLC was well, and the clinical-radiological pretest was quite compatible with the histopathological diagnosis. Despite having a preliminary diagnosis of IPF, histopathological diagnosis was found to be UIP in half of the patients still suspected of having other DPLD. COP was the most common final diagnosis which is determined by the contribution of TBLC to the clinicopathological diagnosis and NSIP was the most common histopathological diagnosis that the clinical-radiological diagnosis has changed.

The clinical utility of TBLC in the DPLD has recently been studied in diagnostic algorithms. In COLDICE study, the diagnostic accuracy of TBLC for the diagnosis of interstitial lung disease was found to be 70.8% compatible with the gold standard SLB [13]. In another study, it was reported 85.7% in DPLD patients by Sindhwani et al. [14] In a study of 32 patients with suspected DPLD, 62.5% of TBLC results showed concordance with clinical diagnosis and the final treatment was pathology guided in 71% (p = 0.027) [15]. In our study, TBLC was diagnostic in 88.5% (n = 54). We found the concordance of clinical-radiological diagnosis (pretest) and histopathological diagnosis (posttest) significant, with a moderate kappa agreement, in IPF patients. When these findings are interpreted, our study showed that although our center has just gained experience, the TBLC procedure was properly implanted in the DPLD diagnostic algorithm and was applied with the correct indication before the recommendations on this subject became clear.

The findings of our study showed that the majority of DPLD patients undergoing TBLC were female and relatively young. In previous studies, it has been reported that male patients are more common [16,17]. Approximately half of our study population were current or active smokers and 13 of our patients had smoking-related ILD. These final diagnoses were RB-ILD (n = 3), UIP (n = 9) and LHH (n = 1). TBLC is recommended as a safe procedure in studies conducted in patients with a diagnosis of smoking-related ILD by Barata et al. [18]. In another study of 24 patients, TBLC was reported as a minimally invasive method for the characterization of small airway diseases (including smoking-related interstitial lung disease) with a low percentage of complications and good diagnostic accuracy [19].

In our study population the main finding detected in pulmonary function tests is decreased DLCO, consistent with the nature of DPLD. Decreased FVC which is a hallmark of IPF and/or decreased FEV_1_ reflecting an obstructive pathology was not demonstrated. In other studies, the study populations of DPLD patients also had diffusion limitations like our findings [13,18].

The complications of TBLC include hemorrhage, pneumothorax, pneumonia, IPF exacerbation, and respiratory failure [20]. Hemorrhage was the most common (n = 10, 16.1%) complication in our study population. The overall complication rate was 2.76% and hemorrhage was 0.92% in a study which 326 TBLC procedures were evaluated [21]. Pneumothorax rates have been reported in a wide range from 0% to over 30% in studies [22]. In our study, the rate of pneumothorax was found to be 6.4%, which is an acceptable rate. No other complications such as infection or acute exacerbation were observed in our study.

Radiological evaluation is an important diagnostic step in DPLD patients. Common predominant categories identified are reticular and nodular changes or diseases associated with diffuse changes in lung density. It is stated that this algorithmic approach will significantly improve the initial interpretation of a wide range of DPLD [23]. The radiological findings in our study were also compatible with these predefined DPLD patterns, such as ground-glass opacification, reticulations, and nodules. The presence of IPF alternative diagnosis patterns such as heterogeneity, apicobasal gradient and upper zone predominance were detected in nearly half of the patients.

The overall diagnostic yield of TBLC was reported between 50%–100% in different studies [24]. In our study, we found TBLC highly diagnostic with a rate of 88.5%. In a study by Kropski et al. IPF was the most frequent prediagnosis (n = 8, 40%) with a diagnostic yield of 80% in 25 patients [25]. Bondue et al. reported a high percentage of granulomatous diseases (34%) and NSIP (20%) in their series of a Belgian population [26]. In our study, most often radiological and clinical-radiological diagnoses were IPF in one-fifth of the patients while after the histopathological evaluation final diagnosis determined by the multidisciplinary team was COP in 22.9%. Three of the six patients who were considered to have IPF in the clinical-radiological pretest evaluation, but whose diagnosis changed in the posttest, were diagnosed with COP. Therefore, as with previous recommendations [27], we suggested performing TBLC if there is clinical suspicion, even if IPF is considered clinical-radiological. On the other hand, since the multidisciplinary definitive diagnosis is usually COP in patients with a clinical-radiological prediagnosis of COP, biopsy can be delayed with suspected COP. In our study, the most common histopathological diagnosis confirmed by TBLC was NSIP, indicating that TBLC is useful in the algorithm for this difficult clinical-radiological diagnosis.

The diagnosis of DPLD requires extensive review by a multidisciplinary panel of experienced pulmonologists, thoracic radiologist, and lung pathologist [1]. Fruchter et al. reported that a definitive clinicopathological consensus diagnosis was possible in 70% of DPLD patients, but it could not be diagnosed in 2% of them [28]. In a study of 150 patients, multidisciplinary evaluation led to change or the initiation of therapy in 55% of cases [29]. In our study, the pretest clinical-radiological diagnosis was often compatible with the multidisciplinary final diagnosis (n = 32, 52.5%).

Our study had some limitations. Our clinic is a tertiary referral clinic and mostly selected patients apply. Our sample was relatively small, and the study was of retrospective design. SLB was applied to only one of the patients whose biopsy results were reported to have normal parenchyma.

In conclusion, the overall diagnostic yield of TBLC was high as with the most common final diagnosis of COP. The pretest clinical-radiological diagnosis was often compatible with the multidisciplinary final diagnosis, reminding a careful multidisciplinary evaluation is critical in the diagnostic algorithm of DPLD. TBLC is useful in the diagnostic algorithm of NSIP which is difficult to diagnose clinical-radiological. Since the histopathological diagnosis was UIP only in half of the patients having a preliminary diagnosis of IPF, we suggest considering TBLC when other DPLDs are suspected despite having a prediagnosis of IPF.

## Figures and Tables

**Table 1:** Demographic and clinical characteristics of the study population.

Age (mean ± SD)	53.3 (±11.5)
Sex, male n(%)	25 (41.0)
Smoking history n (%)	
Current smoker	17 (27.8)
Ex-Smoker	12 (19.6)
Packs year (mean ± SD)	20 (±12.7)
Exposure n (%)	
Occupational	17 (27.4)
Environmental	19 (30.6)
CTD-ILD n (%)	7 (11.3)
Pulmonary function tests (mean ± SD)	
FEV_1_ (L)	2.9 (±0.70)
FEV_1_% predicted	89 (±19.1)
FVC (L)	2.78 (±1.00)
FVC% predicted	90 (±19.4)
FEV_1_/FVC	81 (± 8.45)
DLCO% predicted	61.5 (±16.7)
DLCO/VA% predicted	85.5 (±18.4)
Complications n (%)	
Pneumothorax	2 (3.2)
Hemorrhage	10 (16.1)
^*^Massive hemorrhage	4 (6.4)
Pneumothorax+hemorrhage	2 (3.2)

**Abbreviations:** SD: Standard deviation CTD = Connective tissue disease; DLCO = Single-breath diffusing capacity for carbon monoxide; DLCO/VA = DLCO divided by the alveolar volume; ILD = Interstitial lung disease; FEV_1_= Forced expiratory volume in 1 s; FVC = Forced vital capacity

**Table 2:** Radiological, clinical-radiological, and multidisciplinary final diagnosis of the study population.

Radiological diagnosis		Clinical-radiological diagnosis		Multidisciplinary final diagnosis	
IPF	12 (19.6)	IPF	12 (19.6)	UIP	9 (14.7)
OP	9 (14.7)	COP	10 (16.3)	COP	14 (22.9)
RB	6 (9.8)	RB	2 (3.2)	RB	3 (4.9)
Sarcoidosis	3 (4.9)	Sarcoidosis	6 (9.8)	Granulomatous inflammation	7 (11.4)
NSIP	3 (4.9)	NSIP	2 (3.2)	NSIP	8(13.1)
Other	28 (46.1)	Other	29 (47.9)	Other	20 (33.0)

**Abbreviations:** IPF = Idiopathic pulmonary fibrosis; RB = Respiratory bronchiolitis; COP = Cryptogenic organizing pneumonia; NSIP = Nonspecific interstitial pneumonia; UIP = Usual interstitial pneumonia.

**Table 3:** Clinical-radiological and histopathological diagnosis correlation for idiopathic pulmonary fibrosis patients.

n (%)	Histopathological diagnosis		
Clinical-radiological diagnosis	UIP	Other than UIP	p < 0.001
IPF	6 (50.0)	6 (50.0)	
non-IPF	3 (6.2)	46 (93.8)	

**Abbreviations:** IPF; Idiopathic pulmonary fibrosis.

**Table 4:** Summary of the patients whose diagnosis changed with TBLC.

Diagnosis	Clinical-radiological	Radiological	Histopathological
**Case 1**	IPF	NSIP	OP
**Case 2**	IPF	IPF	Granulomatous inflammation
**Case 3**	IPF	IPF	OP
**Case4**	IPF	no diagnosis	NSIP
**Case 5**	IPF	no diagnosis	OP
**Case 6**	IPF	no diagnosis	CTD-ILD
**Case 7**	LAM	no diagnosis	RBILD
**Case 8**	LAM	no diagnosis	RBILD
**Case 9**	HP	no diagnosis	NSIP
**Case 10**	HP	PPF	NSIP
**Case 11**	HP	no diagnosis	OP
**Case 12**	HP	RBILD	Granulomatous inflammation
**Case 13**	Sarcoidosis	no diagnosis	Vasculitis
**Case 14**	Sarcoidosis	IPF	Pneumoconiosis
**Case 1**5	Vasculitis	no diagnosis	LCH
**Case 16**	Vasculitis	İPF	CTD-ILD
**Case 17**	OP	OP	NSIP
**Case 18**	OP	NSIP	Hemosiderosis
**Case 19**	DIP	no diagnosis	NSIP
**Case 20**	Occupational-ILD	no diagnosis	UIP
**Case 2**1	Drug-Induced	no diagnosis	OP
**Case 22**	CPFE	OP	NSIP

**Abbreviations:** IPF = Idiopathic pulmonary fibrosis; HP = Hypersensitivity pneumonitis; RB = Respiratory bronchiolitis; COP = Cryptogenic organizing pneumonia; NSIP = Nonspecific interstitial pneumonia; DIP = Desquamative interstitial pneumonia; ILD = Interstitial lung disease; LAM = Lymphangioleiomyomatosis; LCH = Langerhans cell histiocytosis; CTD = Connective tissue disease; CPFE = Combined pulmonary fibrosis and emphysema.
